# Hypertrophic Mucoid Degeneration of the Anterior Cruciate Ligament Mimicking a Tear: A Case Report and Diagnostic Pitfall

**DOI:** 10.7759/cureus.108500

**Published:** 2026-05-08

**Authors:** Abdallah Said Abdallah, Ola Messaoud, Zaynab Iraqi Houssaini, Laila Jroundi, Omar El Aoufir

**Affiliations:** 1 Department of Radiology, Ibn Sina University Hospital, Mohammed V University, Rabat, MAR

**Keywords:** acl tear, anterior cruciate ligament (acl), celery stalk sign, diagnostic pitfall, intraosseous cyst, knee mri, mucoid degeneration

## Abstract

Mucoid degeneration (MD) of the anterior cruciate ligament (ACL) is an underdiagnosed condition marked by glycosaminoglycan infiltration within the ligament fibers. Its appearance on magnetic resonance imaging (MRI) can closely mimic an ACL tear, leading to diagnostic errors with major clinical consequences. We report a 54-year-old woman with a 6-month history of chronic knee pain and a sensation of heaviness, without any trauma. Physical examination showed a mildly positive anterior drawer test, interpreted as pain-limited rather than indicative of true instability, with a negative Lachman test. MRI revealed a thickened ligament with diffuse high signal intensity on proton density fat-saturated sequences, a celery stalk appearance on sagittal images, and a markedly thickened, crescent-shaped axial appearance of the hypertrophied ligament filling the intercondylar notch. The fibers appeared preserved and parallel to the Blumensaat line. Additional findings included near-complete filling of the intercondylar notch, intraosseous cysts at both the femoral and tibial ACL footprints, a Stoller grade III signal in the anterior horn of the lateral meniscus consistent with a meniscal tear, and a small joint effusion. The patient was managed conservatively with non-steroidal anti-inflammatory drugs and physiotherapy, with significant pain reduction and near-complete recovery of knee extension at six to eight weeks of follow-up. This case illustrates the imaging features that distinguish mucoid degeneration from ligamentous rupture on MRI, since misdiagnosis can lead to unnecessary surgery.

## Introduction

The anterior cruciate ligament (ACL) is the primary ligamentous restraint preventing anterior translation of the tibia relative to the femur and is essential for rotational knee stability; any structural disorder of this ligament can therefore mimic the clinical presentation of a tear. The ACL is the most frequently injured ligament of the knee, and magnetic resonance imaging (MRI) is the reference standard for its evaluation. Among the less recognized pathologies affecting this structure, mucoid degeneration (MD) remains frequently underdiagnosed. The condition results from the accumulation of glycosaminoglycans (mucopolysaccharide molecules of the extracellular matrix) within the collagenous bundles of the ligament, leading to hypertrophy and diffuse signal alteration on MRI [1,2]. In our case, MD presented with marked ligamentous hypertrophy, a feature that can cause mechanical impingement within the intercondylar notch (the bony groove between the femoral condyles housing the cruciate ligaments). It's reported MRI prevalence ranges from 1.8% to 5.3%, with a predilection for patients over 40 years [3,4]. Until recently, no standardized MRI classification existed; the SHRINK classification was proposed by Saran et al. in 2026 to address this gap [5]. Although not yet externally validated by independent cohorts, it provides the first systematic framework for stratifying mucoid degeneration based on signal pattern, hypertrophy, and associated cystic changes. The diffuse high signal of a thickened ACL can closely resemble a partial or complete tear, creating a well-documented diagnostic pitfall [6]. This distinction is clinically critical because management differs fundamentally: tears often require surgical reconstruction, whereas MD is usually managed conservatively or with arthroscopic debridement [7,8]. Misdiagnosis, therefore, carries a risk of unnecessary and potentially harmful surgery. We report the case of a 54-year-old woman in whom the MRI appearance closely mimicked a ligamentous tear, while clinical examination remained equivocal.

## Case presentation

A 54-year-old woman with no significant past medical history presented with a 6-month history of progressive right knee pain and a sensation of heaviness. She reported no history of trauma, mechanical instability, or locking--an important clinical feature distinguishing mucoid degeneration from traumatic ACL tear in middle-aged patients. Physical examination revealed a mildly positive anterior drawer test with a negative Lachman test. The mildly positive anterior drawer test was interpreted as pain-limited rather than indicative of true mechanical instability, consistent with the negative Lachman test and the absence of functional instability. Range of motion was mildly limited in terminal extension, with an estimated extension deficit of approximately 5 to 10 degrees compared to the contralateral side. This finding was considered consistent with mechanical impingement of the hypertrophied ACL within the intercondylar notch.

**Figure 1:**
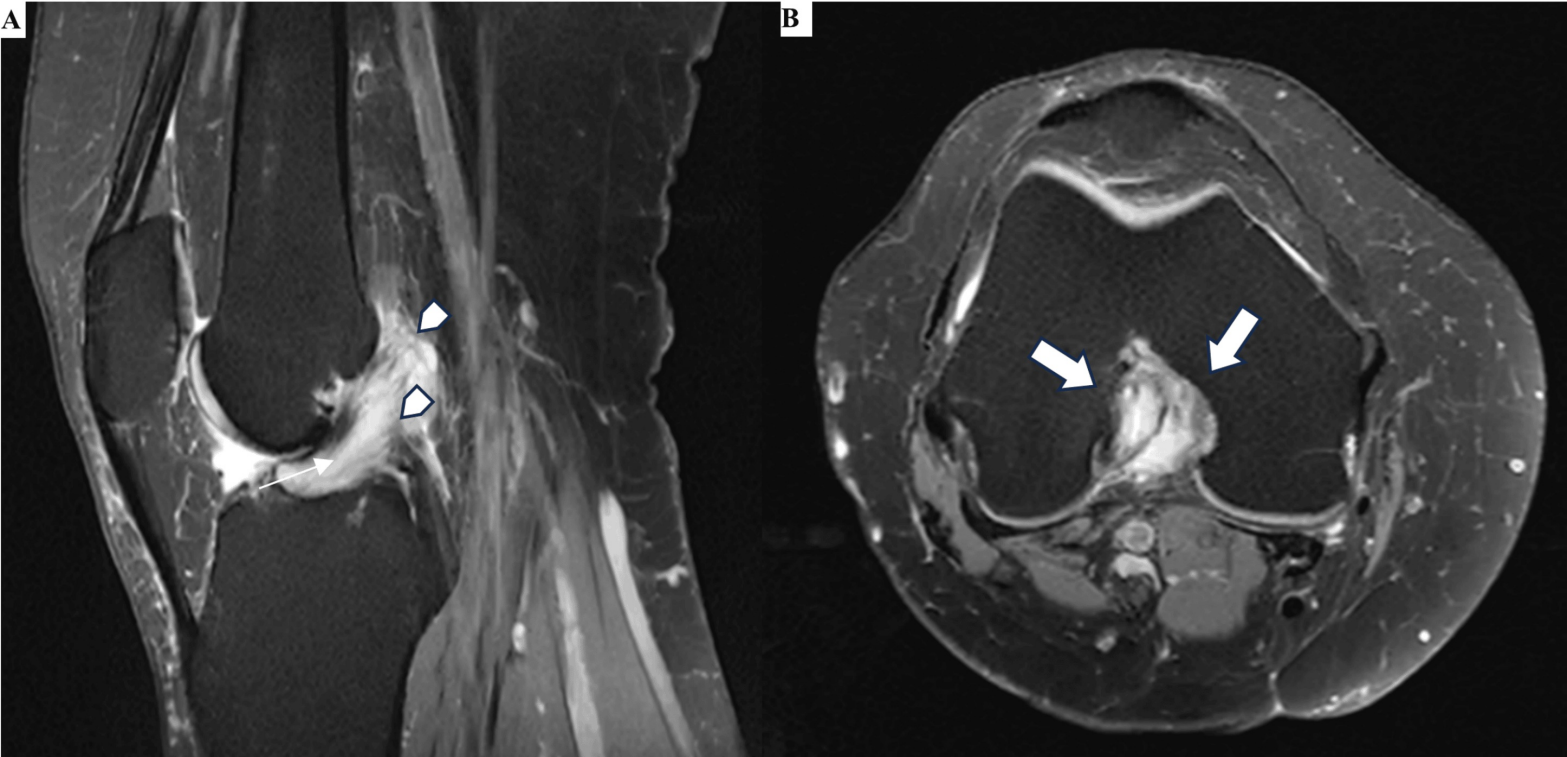
MR images of the right knee showing direct signs of mucoid degeneration of the anterior cruciate ligament MR images of the right knee (A) Sagittal PD-FS image: celery stalk sign with preserved fibers (arrowheads) and interstitial high-signal material (arrow), parallel to the Blumensaat line. (B) Axial PD-FS image: crescent-shaped appearance of the hypertrophied ACL (arrows) filling the intercondylar notch. PD-FS: proton density fat-saturated; ACL: anterior cruciate ligament

A knee MRI was performed on a 1.5T system using a dedicated knee coil. The protocol comprised proton density fat-saturated (PD-FS) sequences in three orthogonal planes (sagittal, coronal, axial; TR/TE 2260/35 ms) and a sagittal T1-weighted sequence (TR/TE 600/15 ms), with a slice thickness of 3 mm. The ACL was markedly thickened with diffuse high signal intensity on PD-FS sequences, but the ligament fibers were preserved and parallel to the Blumensaat line (the radiographic line along the roof of the intercondylar notch), with a characteristic celery stalk appearance (Figure 1A). On axial sections, the hypertrophied ligament adopted a crescent-shaped appearance, nearly filling the intercondylar notch (Figure 1B).

**Figure 2:**
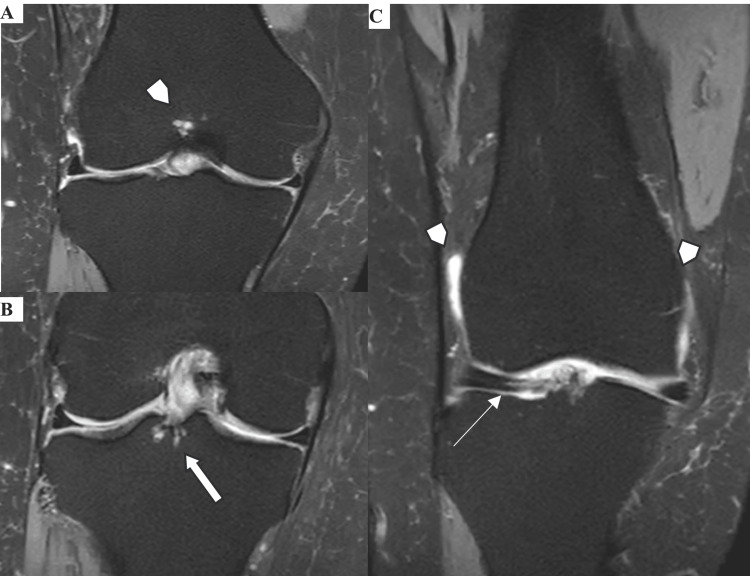
Associated MRI findings of mucoid degeneration of the anterior cruciate ligament Coronal PD-FS images (A) Intraosseous cyst at the femoral ACL footprint (arrowhead). (B) Intraosseous cyst at the tibial ACL footprint (arrow). (C) Stoller grade III signal in the anterior horn of the lateral meniscus consistent with a meniscal tear (arrow), with small joint effusion (arrowheads). PD-FS: proton density fat-saturated

Additional findings included well-defined hyperintense intraosseous cysts on PD-FS sequences at both the femoral and tibial ACL footprints (Figures 2A-2B), a Stoller grade III signal in the anterior horn of the lateral meniscus consistent with a meniscal tear, with a small joint effusion (Figure 2C). The meniscal lesion may have contributed to the patient's pain, but the dominant complaint of deep posterior pain and a sensation of heaviness was more consistent with intercondylar impingement from ACL hypertrophy than with lateral joint-line pain. No mechanical locking was reported. No secondary signs of ACL injury were identified: no bone contusions, no deep lateral femoral notch, and no anterior tibial translation.

Based on these imaging findings and the absence of secondary signs of ligamentous injury, a diagnosis of mucoid degeneration was made. Arthroscopic confirmation was not performed, as surgery was not clinically indicated. The patient was managed conservatively with non-steroidal anti-inflammatory drugs and a structured physiotherapy program focusing on range-of-motion recovery and muscle strengthening. At clinical follow-up approximately 6 to 8 weeks later, she reported significant pain reduction with near-complete recovery of knee extension and no functional instability. Given this favorable clinical evolution, no surgical intervention was indicated.

## Discussion

First described by Kumar et al. in 1999 under the term "mucoid cystic degeneration of the cruciate ligament" in a 35-year-old man with an intraligamentous ACL mass confirmed histologically [1], mucoid degeneration (MD) has since been increasingly recognized but remains underreported [4]. MD can be easily misinterpreted as a partial or complete ACL tear. Unlike tears, which show fiber discontinuity, abnormal orientation, and secondary signs, such as bone contusions, a deep lateral femoral notch, and anterior tibial translation, exceeding five millimeters [9,10], MD preserves ligament continuity [2,11]. The MRI hallmark is a thickened ligament with diffuse high signal, known as the celery stalk sign. In our patient, fibers remained individually discernible and parallel to the Blumensaat line. On axial sections, the hypertrophied ACL adopted a crescent-shaped appearance filling the intercondylar notch, a morphologic clue reflecting marked ligament hypertrophy. To avoid terminological confusion, this finding should be distinguished from the radiographic subchondral crescent sign of femoral head osteonecrosis, which describes a different entity at a different anatomic site. Beyond these direct ligamentous signs, three positive criteria support the diagnosis of mucoid degeneration in our case: preserved multiplanar fiber continuity, absence of secondary signs of ACL injury, and bilateral footprint cysts. Bergin et al. reported such cysts in the majority of MD cases [3], and Cilengir et al. showed that a tibial intraosseous cyst increased the probability of MD 41.2-fold compared with ACL sprain [12], a particularly strong discriminator in cases where ligamentous appearance alone remains ambiguous. Makino et al. further confirmed histologically that such cysts were present only in patients with definitive MD, not in cases of misdiagnosis [13]. Their bilateral presence in our case strongly supports the diagnosis.

Although MD classically lacks ligamentous instability [2,7], our patient showed a mildly positive anterior drawer test with a negative Lachman test, interpreted as pain-limited anterior translation rather than true insufficiency. In chronic settings, both tests show comparable sensitivity (92% and 95%, respectively) [14], although Celikyay et al. found that 14% of patients with MD had anterior tibial translation greater than five millimeters [9]. The near-complete filling of the intercondylar notch is a well-known consequence of ligament hypertrophy, with Cha et al. reporting a reduced notch width in these patients (16.0 vs 19.3 mm) [15]. This impingement accounts for the terminal extension deficit observed in our patient [2].

Several diagnostic pitfalls deserve emphasis. The diffuse high signal of a thickened ACL can be mistaken for an interstitial partial tear [6]. Multiplanar analysis, demonstrating preserved fiber continuity in all planes, is essential to avoid this error [2,11]. In the absence of a trauma history, particularly in patients over 40 years, the diagnostic suspicion should systematically shift toward MD rather than a traumatic tear, especially when the ligament shows hypertrophy with preserved continuity [2,7]. Imaging-based diagnosis is adequate when characteristic features are present and clinical instability is absent [2,7]. Applied to our patient, the findings correspond to Category 5 of the recently proposed SHRINK classification [5], which designates MD with associated bony abnormalities (subchondral edema or cystic changes)--an uncommon presentation, observed in only 2% of cases in the original cohort. Conservative management remains the first-line approach, with partial debridement reserved for symptomatic cases [7].

Some limitations of this case report should be acknowledged. The diagnosis of mucoid degeneration was established based on characteristic MRI features without arthroscopic or histological confirmation, since surgery was not clinically indicated and the patient evolved favorably under conservative management. The lack of pathological correlation prevents definitive validation, even though imaging-based diagnosis is considered acceptable in this clinical context [2,7]. The short-term follow-up of six to eight weeks does not allow assessment of long-term evolution, and the inherent constraints of a single-case observation limit the generalizability of these findings.

## Conclusions

Mucoid degeneration of the ACL can closely mimic a ligamentous rupture on MRI, with clinical examination occasionally showing equivocal findings such as a mildly positive anterior drawer test attributable to pain-limited muscular guarding. Recognition of the celery stalk sign, preserved fiber parallelism, the crescent-shaped axial appearance of the hypertrophied ligament, and footprint cysts, combined with the absence of secondary signs of injury, allows accurate differentiation and prevents unnecessary surgery. This entity should be included in the differential diagnosis of any thickened, hyperintense ACL on fluid-sensitive sequences, especially in middle-aged patients without trauma.
